# Spatial Representations in Local Field Potential Activity of Primate Anterior Intraparietal Cortex (AIP)

**DOI:** 10.1371/journal.pone.0142679

**Published:** 2015-11-10

**Authors:** Sebastian J. Lehmann, Hansjörg Scherberger

**Affiliations:** 1 Deutsches Primatenzentrum, 37077, Göttingen, Germany; 2 Institute of Neuroinformatics, University of Zürich and ETH Zürich, CH-8057, Zürich, Switzerland; 3 Department of Biology, University of Göttingen, D-37077, Göttingen, Germany; 4 Department of Physiology & Pharmacology, Robarts Research Institute, University of Western Ontario, London, ON N6A 5B7, Canada; Centre de Neuroscience Cognitive, FRANCE

## Abstract

The execution of reach-to-grasp movements in order to interact with our environment is an important subset of the human movement repertoire. To coordinate such goal-directed movements, information about the relative spatial position of target and effector (in this case the hand) has to be continuously integrated and processed. Recently, we reported the existence of spatial representations in spiking-activity of the cortical fronto-parietal grasp network (Lehmann & Scherberger 2013), and in particular in the anterior intraparietal cortex (AIP). To further investigate the nature of these spatial representations, we explored in two rhesus monkeys (*Macaca mulatta*) how different frequency bands of the local field potential (LFP) in AIP are modulated by grip type, target position, and gaze position, during the planning and execution of reach-to-grasp movements. We systematically varied grasp type, spatial target, and gaze position and found that both spatial and grasp information were encoded in a variety of frequency bands (1–13Hz, 13–30Hz, 30–60Hz, and 60–100Hz, respectively). Whereas the representation of grasp type strongly increased towards and during movement execution, spatial information was represented throughout the task. Both spatial and grasp type representations could be readily decoded from all frequency bands. The fact that grasp type and spatial (reach) information was found not only in spiking activity, but also in various LFP frequency bands of AIP, might significantly contribute to the development of LFP-based neural interfaces for the control of upper limb prostheses.

## Introduction

Reach-to-grasp movements are a key asset for everyday interactions with our environment. To generate such actions, both humans and non-human primates need to combine two different kind of upper limb movements: a reach action, bringing the hand (or effector) closer to a given object, and a grasping action to manipulate the object in a highly versatile fashion. To realize this process, sensory, and in particular visual, information about the relative spatial position of a reach-to-grasp target and of the effector has to be continuously processed and integrated. However, the question is still open how the brain achieves this important visuo-motor transformation.

Several cortical areas have been shown to represent visuo-motor features during the planning and execution of grasp movements. In the primate parietal cortex the anterior intraparietal area (AIP) has been shown to be relevant for hand shaping [1–5], representation of visual features of grasp targets [6–8], and the encoding of wrist orientation [9]. Recently, we demonstrated that single neurons in AIP not only encode grip type, but also the target position in retinotopic coordinates [10]. Anatomically, AIP provides reciprocal connections to other parietal and premotor areas, in particular to the parietal areas PRR, V6A, and LIP and to the ventral premotor cortex (area F5) [5, 11–13], which renders AIP a crucial area for the visuo-motor information processing for hand grasping.

Local field potentials (LFPs) are a valuable source for understanding brain processes. They provide a potential link between intra-cortical recordings and non-invasive methods like fMRI or EEG [14–16]. LFPs are often interpreted as a summation signal of inhibitory and excitatory dendritic potentials, reflecting information about network states and intra-cortical processing around the recording site [14, 15, 17, 18].

LFPs encode motor related features in various visuo-motor cortical areas. For example, eye movements are represented in LFP activity in the lateral intraparietal area [19], and reach movement directions could be decoded from LFPs in the parietal reach region [18] as well as in primary motor cortex [20–22]. LFP signals are relatively stable and easier to record than spiking activity, especially over longer time periods, and they might therefore be of particular interest for the development of brain machine interfaces [17, 18, 23].

Only few studies have investigated LFP signals in the ‘classical’ hand grasping areas, as mentioned above. In AIP, LFP signals have been demonstrated to encode the reach direction, but this representation was mostly present during movement execution [24]. In premotor area F5, LFP selectivity was reported for grasp type during movement execution [25] and a more recent study demonstrated the possibility of using LFPs to robustly decode reach and grasp kinematics in M1 and PMv [23]. However, relatively little is known about LFP coding properties during grasp action planning or preparation.

Following up on a previous study that demonstrated that single units in AIP represent grip type and spatial information [10], we specifically investigated here how LFP activity in AIP is modulated during the planning and execution of reach-to-grasp movements, in particular with respect to variations of grip type and of spatial factors like target and gaze position.

## Materials & Methods

### Ethics statement

All procedures and animal care were conducted in accordance with the guidelines for the care and use of mammals in neuroscience and behavioral research (National Research Council, 2003), and were in agreement with Swiss, German, and European laws governing animal care. Authorization for conducting this experiment was delivered by the Veterinary Office of the Canton of Zurich, Switzerland (permit no. 78/2007) and the Animal Welfare Division of the Office for Consumer Protection and Food Safety of the State of Lower Saxony, Germany (permit no. 032/09). Monkey handling also followed the recommendations of the Weatherall Report of good animal practice.

Two female, captivity-born rhesus macaque monkeys (*Macaca mulatta*) participated in this study (animals P and S; weight 4.5 and 5.5 kg, respectively). They were pair-housed in a spacious cage (well exceeding legal requirements) and maintained on a 12-hour on/off lightning schedule. Housing procedures included an environmental enrichment program with access to toys, swings, and hidden treats (e.g., seeds in sawdust). Monkeys had visual and auditory contact to other monkeys. They were fed on a diet of enriched biscuits and fruits. Daily access to fluids was controlled during training and experimental periods to promote behavioral motivation. All surgical procedures were performed under anesthesia, and all efforts were made to minimize post-surgical pain or suffering. Institutional veterinarians continually monitored animal health and well-being.

### Experimental Setup

**Fig 1:**
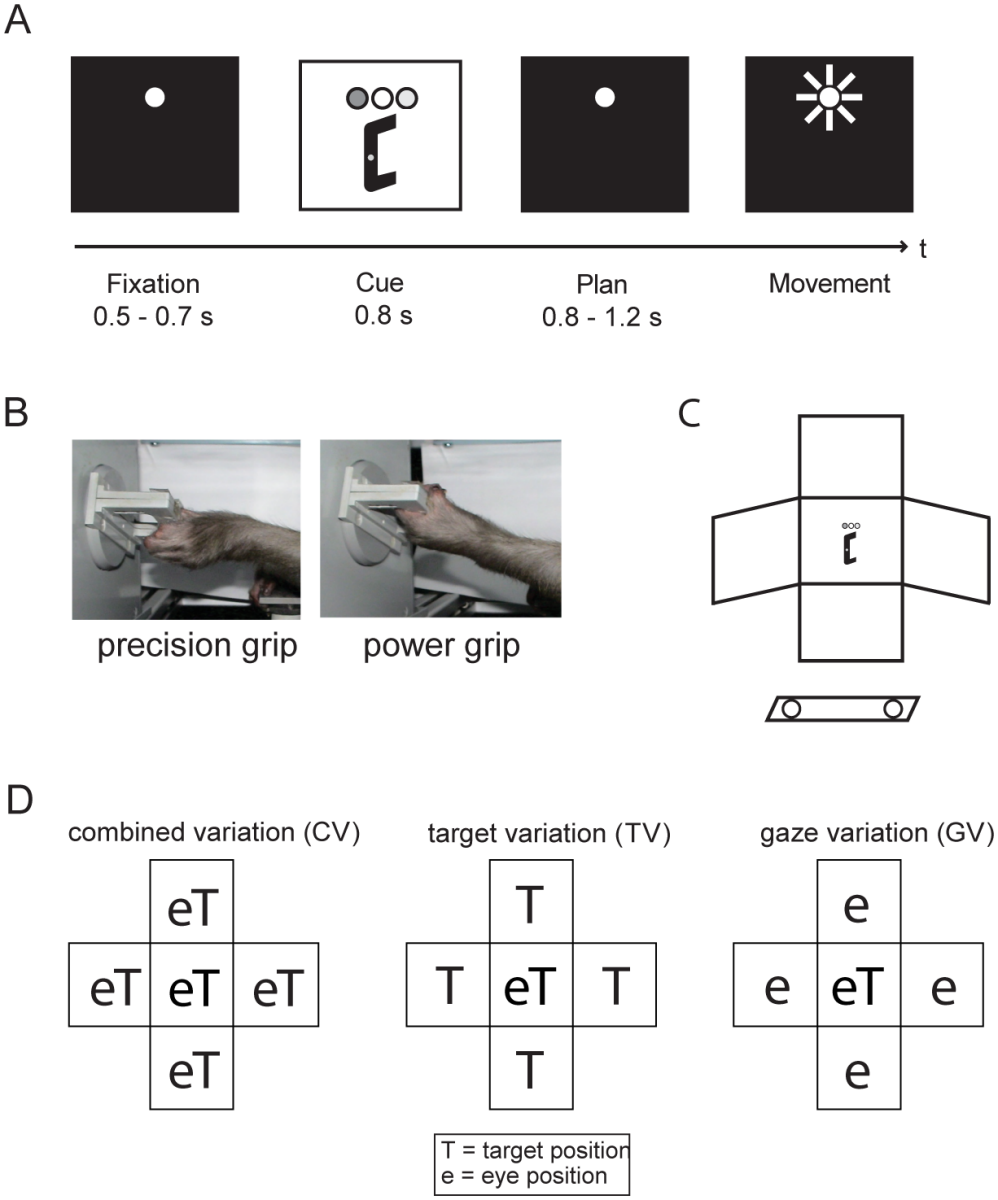
Behavioral task design. A. Delayed reach-to-grasp task with the epochs fixation, cue, planning, and movement. Trials were initiated by placing both hands on hand rest sensors and fixating a red LED. After 500-700ms (fixation epoch), the instruction which grip type to use (precision or power grip, indicated by the color of a second LED) and the target position (by illumination) were revealed (cue epoch). After a planning epoch of 800-1200ms, a blink of the fixation LED instructed the monkey to grasp the target in the dark, while maintaining eye fixation. After successful termination, the animal got rewarded with a fixed amount of liquid. B. The target was grasped with either a precision grip (left) or a power grip (right photo). C. Schematic of the reach-to-grasp experimental setup. After placing both hands on the sensors in front of the body (circles), the monkeys had to grasp for the target positioned in front of them. D. Schematic view of the possible spatial variations. Target (T) and gaze positions (e for eye) were systematically varied, resulting in the subtasks CV (combined variation), with target and gaze presented in five joint positions, TV (target variation), with gaze position in the center, and GV (gaze variation), with target position in the center.

Animals were sitting in a primate chair with the head rigidly fixed. A grasp target was positioned in front of the animal, which consisted of a handle that could be grasped with two different grip types, either a precision grip (using index finger and thumb in opposition) or a whole-hand power grip. The horizontal and vertical position of the target, and therefore the reach direction, was varied by shifting it in front of the animals with two motors (Fig 1C). In addition, the eye fixation position was varied by addressing different LEDs, either in combination with variation of target position, or independent of it. Spatial target position was changed automatically in the dark, in between trials. Eye position was monitored with an optical eye tracking system (ET-49B; Thomas Recording, Germany). Touch sensors in front of the animal’s hips monitored the resting position of both hands. Animal behavior and stimulus parameters were controlled with a time resolution of 5 ms using custom-written code (Lab-View Realtime, National Instruments).

### Task Paradigm

Monkeys performed a delayed reach-to-grasp task after being instructed to grasp a target with either a power grip or a precision grip. A trial started when the animal placed both hands on the hand-rest sensors and fixated a red LED (Fig 1A). This fixation epoch (500–700 ms) was followed by a cue epoch (fixed length: 800 or 1000 ms), in which an additional LED next to the fixation position instructed the animal which grasp type to use (green LED: power grip, orange LED: precision grip; Fig 1B depicts the grip types). In addition, the grasp target was illuminated, revealing the handle position in space. In the following planning epoch of variable length (800–1200 ms) only the fixation light was visible. Finally, a short blink of the fixation light (“go” cue) instructed the animal to reach and grasp the target (movement epoch) with the left arm (contralateral to the recording chamber). In order to separate the representation of visual stimuli from motor planning and execution, planning and movement epochs were in complete darkness (except the fixation LED). Correctly executed trials were rewarded with a fixed amount of juice, followed by an intertrial interval before the start of the next trial.

During the intertrial interval, target and gaze position were systematically varied (Fig 1C illustrates the experimental setup). The resulting task conditions can be grouped in different subtasks. (1) Combined gaze-and-reach variation (CV), in which target and gaze position were varied together and tested at the center, left, right, top, and bottom position of the workspace (Fig 1D, left). (2) Target variation (TV): gaze position was located at the center of the workspace while target position was either at the center or at the left, right, top, or bottom position (Fig 1D, middle). (3) Gaze variation (GV): target position was located at the center of the workspace while gaze position was varied between the center, left, right, top, and bottom position (Fig 1D, right). In combination with both grip types (power and precision grip), this led to a set of 26 task conditions that were presented pseudo-randomly interleaved, with typically 10 trials per condition.

### Surgical procedures and MRI scans

A titanium head post was secured in a dental acrylic head cap and a custom-made, oval-shaped recording chamber was implanted over the right hemisphere to provide access to AIP. Structural magnetic resonance image (MRI) scans of the brain and skull were obtained from each animal to guide the chamber placement, and again to register the coordinates of the chamber with the cortical structures. AIP was defined as the rostral part of the lateral bank of parietal sulcus [13]. Details of the surgical procedures and MRI scans have been described before [10].

### Recordings

A multiple electrode system (5-channel Mini-Matrix; Thomas Recording, Germany) was used to record cortical activity with quartz-glass-coated platinum/tungsten electrodes (impedance 1–2 MOhm at 1 kHz). Neural signals were amplified (400x), digitized with 16-bit resolution at 30kS/s using a Cerebus Neural Signal processor (Blackrock Microsystems, USA), and saved together with the behavioral data.

### Data analysis

All data was analyzed with Matlab (Mathworks, USA). LFP activity was extracted from the raw signal using a low-pass filter (1–200 Hz), resampled to 1kHz, and cut into behavioral trials. For systematic LFP analysis, frequency decomposition was performed using the “Chronux” signal processing toolbox for Matlab [26]. We performed multi-taper analysis in a sliding window for each trial (window size 300 ms, steps of 50 ms, 3 tapers, time-bandwidth product W = 6.67 Hz (given K = 2NW-1). To visualize the results of the multi-taper spectral analysis, spectrograms *P* from each recording site were averaged over all trials and normalized for each processed frequency band by dividing by the averaged baseline activity *P*_0_ (-2 s to -1.5 s prior to fixation onset). These spectrograms were then averaged across all sites for each animal, and presented as a normalized population spectrogram in logarithmic units (i.e., in decibels): *P*_*db*_ = 10 lg(*P*/*P*_0_).

Further analysis was performed after averaging the LFP spectral power within the following four frequency bands: the slow band (1–13 Hz), the beta band (13–30 Hz), the low gamma band (30–60 Hz), and the high gamma band (60–100 Hz). To test for significant tuning for grip type and spatial position in each task epoch (fixation, cue, planning, movement), spectral power was averaged within each frequency band and task epoch for each trial, and the resulting data points were subjected to a two-way ANOVA across trials (factors grip type and spatial condition, p<0.05). Preferred grip type was defined as the grasp with the higher averaged spectral power separately for each site, epoch, and frequency band.

In order to define a tuning onset for grip type and position, the same analysis (two-way ANOVA, factors grip type and position, p < 0.05) was repeated for each frequency band using a sliding window (size 300 ms, steps 50 ms). Tuning onset was defined as the first of at least five consecutively tuned windows in a row.

Furthermore, to identify the components that contribute to positional tuning, we modeled the averaged LFP power *P* of a given frequency band and task epoch as a stepwise linear model with factors grip type (GT), target position (T), and gaze position (G):

P  =   a  +   gt * GT   +   t * T   +   g * G (1)

Since the spatial factors T and G each have a horizontal (x) and a vertical (y) component, a more detailed description of the model is given by:

P  =   a  +   gt * GT      +      tx * Tx    +   ty * Ty     +      gx * Gx    +   gy * Gy (2)

The stepwise linear fit started with the constant model *P = a* and added additional components in a stepwise fashion until no further significant improvements could be achieved (MATLAB function: *stepwisefit*, p<0.05).

A given site was considered modulated by a particular spatial factor (T or G) if the model contained at least one significant horizontal or vertical component. This allowed us to further categorize each recording site according to the significant modulation of these factors in each frequency band and epoch. The coefficients of this linear regression analysis were used to calculate a tuning direction difference as the angle between the vectors $\text{t }=\;\left(\begin{array}{c} \;tx\\ \;ty \end{array}\right)$ and $\text{g }=\;\left(\begin{array}{c} \;gx\\ \;gy \end{array}\right)$, which becomes 0 deg for parallel and 180 deg for opposing vectors. Furthermore, a length contrast (LC) between these vectors was defined as

$$\text{LC }=\frac{\;\left\|\;\text{t}\;\right\|\;-\;\left\|\;\text{g}\;\right\|\;}{\left\|\;\text{t}\;\right\|\;+\;\left\|\;\text{g}\;\right\|},$$ (3)

such that LC = 0 indicates equal length of both vectors.

Furthermore, an LFP site was considered to encode the spatial position in a retinotopic fashion (or short: retinotopically) if it fulfilled the following three criteria: (i) it was significantly modulated by both target *and* gaze position (as revealed in the stepwise linear regression), (ii) it showed a LC between -0.33 and +0.33 (i.e., target and gaze vector length differed by less than a factor 2), and (iii) the angular difference between t and g was larger than 135 degree (i.e., t and g pointed in approximately opposite directions). These assumptions are reasonable, as for retinotopic coding *t = -g*, and hence *f* = *a* + *gt* * *GT* + *t* * (*T* − *G*).

Finally, a decoding simulation was performed to predict grip type and the spatial factors from the LFP population activity using a maximum likelihood estimator. This decoding analysis was based on the population of sequentially recorded LFP sites, which were here assumed to be recorded simultaneously; hence the term “simulation”. For every trial, spectral power in each frequency band was averaged across each task epoch. Decoding was then performed on a “pruned” database that included only those recording sites and frequency bands that were significantly modulated by the given factor (grip type or position; one-way ANOVA, p<0.05); for details of the decoder see [27] or [18]. Decoding performance was defined as the percentage of correctly decoded conditions from 100 simulated repetitions, in which from each LFP site one trial was randomly selected for decoding while all remaining trials were used to train the decoder. This process was iterated 100 times to obtain performance statistics (mean and standard deviation).

## Results

Two female macaque monkeys (*Macaca mulatta*, animals P and S) performed a delayed reach-to-grasp task, in which the animal grasped a vertical handle (target) in front of it with either a precision grip or power grip (for details see Material and Methods section). Importantly, between trials both the target position and the gaze position were systematically varied in space. Target and gaze could be localized in a combined spatial position (combined variation task (*CV*), Fig 1D) or they could be separated from each other; i.e., either the target position was varied while the gaze position remained centered (target variation task, *TV*), or gaze position was varied with centered target position (gaze variation task, *GV*). In combination of both grip types, this resulted in a set of 26 task conditions, which were presented pseudo-randomly interleaved. Using this paradigm, we recorded LFP activity from a total of 246 sites in AIP (animal P: 147 sites; animal S: 99 sites).

### Task related modulation of LFP activity

**Fig 2:**
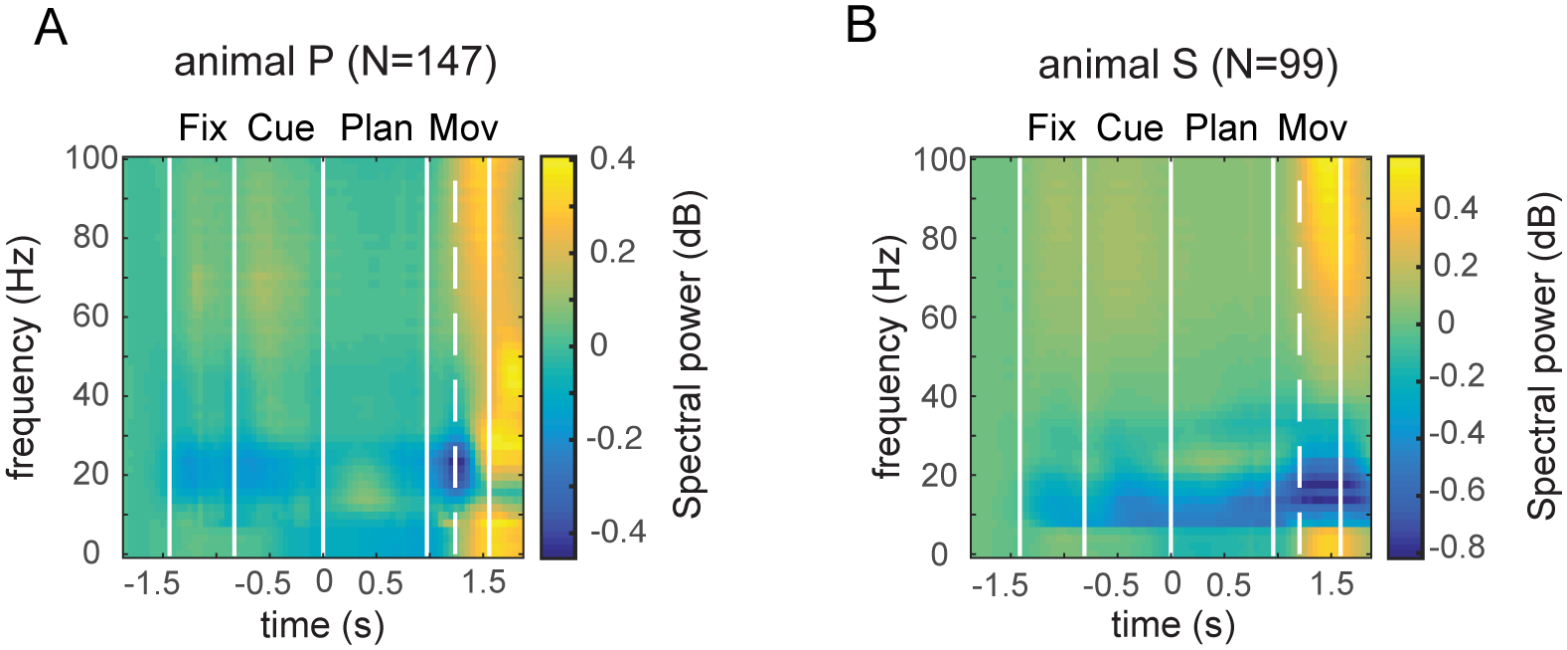
LFP spectrograms. Averaged LFP spectrograms for animal P (A) and animal S (B), normalized to the baseline epoch (prior to fixation onset), and averaged across all recording sites (147 sites in animal P, 99 sites in animal S). Vertical solid lines indicate on- and offset of the fixation, cue, planning, and movement epochs; dashed line indicates movement onset (i.e., hand rest sensor release). Sharp transitions in the slow band are due to the applied multi-taper spectral analysis (time-frequency bandwidth 6.67Hz; see Methods).

To analyze the tuning properties of the LFP for different frequencies, we grouped the LFP spectrogram into the following frequency bands: *slow band* (1–13 Hz), *beta band* (13–30 Hz), *low gamma* band (30–60 Hz), and *high gamma* band (60–100 Hz). We found in both animals task related modulation of the LFP. Fig 2 illustrates LFP spectrograms of both animals, averaged across all recording sites. Spectral power was highest at movement execution in the slow band (1–13 Hz) and both gamma bands (30–60 Hz, 60–100 Hz). In contrast, before movement execution the clearest modulation of LFP power was present in the beta band (13–30 Hz), which showed a decrease of spectral power at the beginning of the task (fixation and cue epoch), followed by an activity increase during the planning epoch, and again a decrease of LFP power during movement execution. In the slow band (1-13Hz) a general decrease in activity was found after cue onset and in the planning epoch, which was followed by an increase during movement execution.

### LFP selectivity

#### Grip type tuning

**Fig 3:**
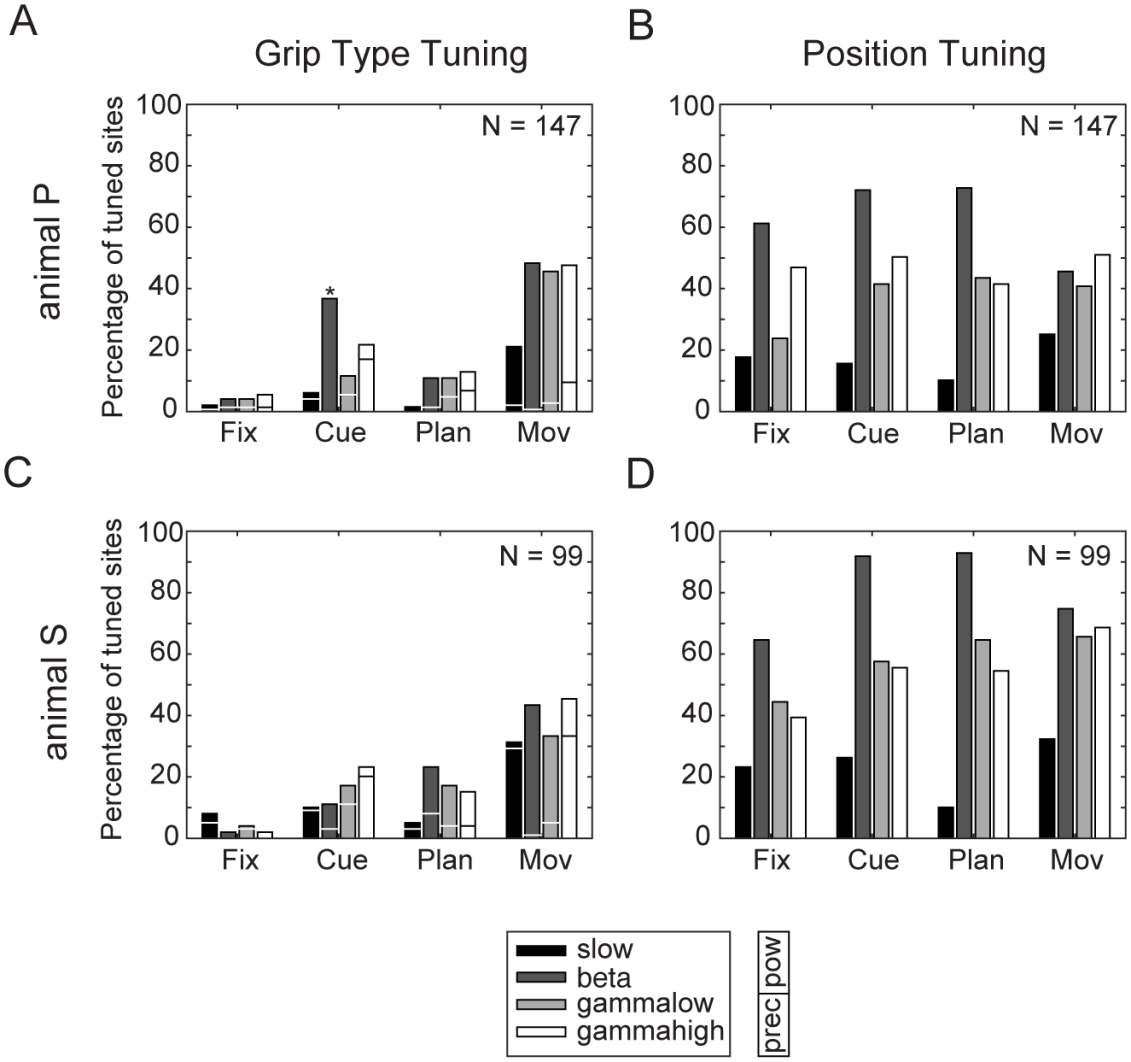
Grip type and position tuning of LFP frequency bands. Percentage of sites tuned (two-way ANOVA, p < 0.05) for the factors grip type (A,C) and position (B,D) for animal P (A,B) and animal S (C,D). Tuning percentages are shown for the slow frequency band (black bars), beta band (dark grey), low gamma band (light grey) and high gamma band (white). Horizontal lines inside bars in A,C indicate fraction of sites tuned for precision (bottom) and power grip (top). Asterisk in Fig 3A indicates preference of 100% for power grip for the beta band during the cue epoch in animal P.

We analyzed the spectral selectivity of the LPF for grip type and spatial positions, separately for the four frequency bands (two-way ANOVA with factors grip type and position, p < 0.05). As shown in Fig 3A, we found significant grip type tuning effects after cue onset in most bands. In both animals, the biggest grip type tuning effects were found in the movement epoch, being relatively consistent across bands (with an average of 41% of sites across frequency bands significantly tuned for grip type in animal P [*slow*: 21%, *beta*: 48%, *gamma low*: 46%, *gamma high*: 48%) and of 38% in animal S (*slow*: 31%, *beta*: 43%, *gamma low*: 33%, *gamma high*: 45%; Fig 3A and 3C).

In addition, we determined the preferred grip type of the LFP spectral power separately for each frequency band: the grip type (power vs. precision grip) with the higher averaged power was taken as the “preferred” grip type. Even though the number of sites being tuned for grip type within the movement epoch was similar for both animals, there were clear differences in the preferred grip type between them. For animal P, a strong majority of grip-type modulated sites showed a preference for power grips during the movement epoch (91% of all sites across frequencies; *slow band*: 90%, *beta band*: 99%, *low gamma band*: 94%, *high gamma band*: 80%, see: Fig 3A). However, in animal S, individual frequency bands also showed strong preferences for a certain grip type, but preferences varied across frequency bands: whereas the majority of modulated sites of the beta and low gamma band showed a preference for power grip, most sites in the slow band and the high gamma band were tuned for precision grip (preferences for power grip in animal S: *slow band*: 6%, *beta band*: 98%, *low gamma band*: 85%, *high gamma band*: 27%).

#### Spatial Tuning

In contrast to the strongly increasing representation of grip type toward movement execution, the representation of spatial information in the LFP spectrum was more constant across different task epochs. This was true for all frequency bands in both animals (Fig 3B and 3D). However, the highest percentage of spatially tuned recordings sites was found in the beta band, with high levels in all task epochs and peaks in the cue and planning epoch (*fix*: 61% in animal P / 65% in animal S; *cue*: 72% / 92%; *plan*: 73% / 93%; *movement* 46% / 75%). Interestingly, in both animals, the slow band showed a decrease in tuning during the planning epoch, before increasing again during movement. Similarly, the high gamma band showed a slight increase of modulated sites at the time of movement start. Altogether, we confirmed a strong selectivity of the LFP for grip type and for spatial position in both animals.

### Tuning onset

**Fig 4:**
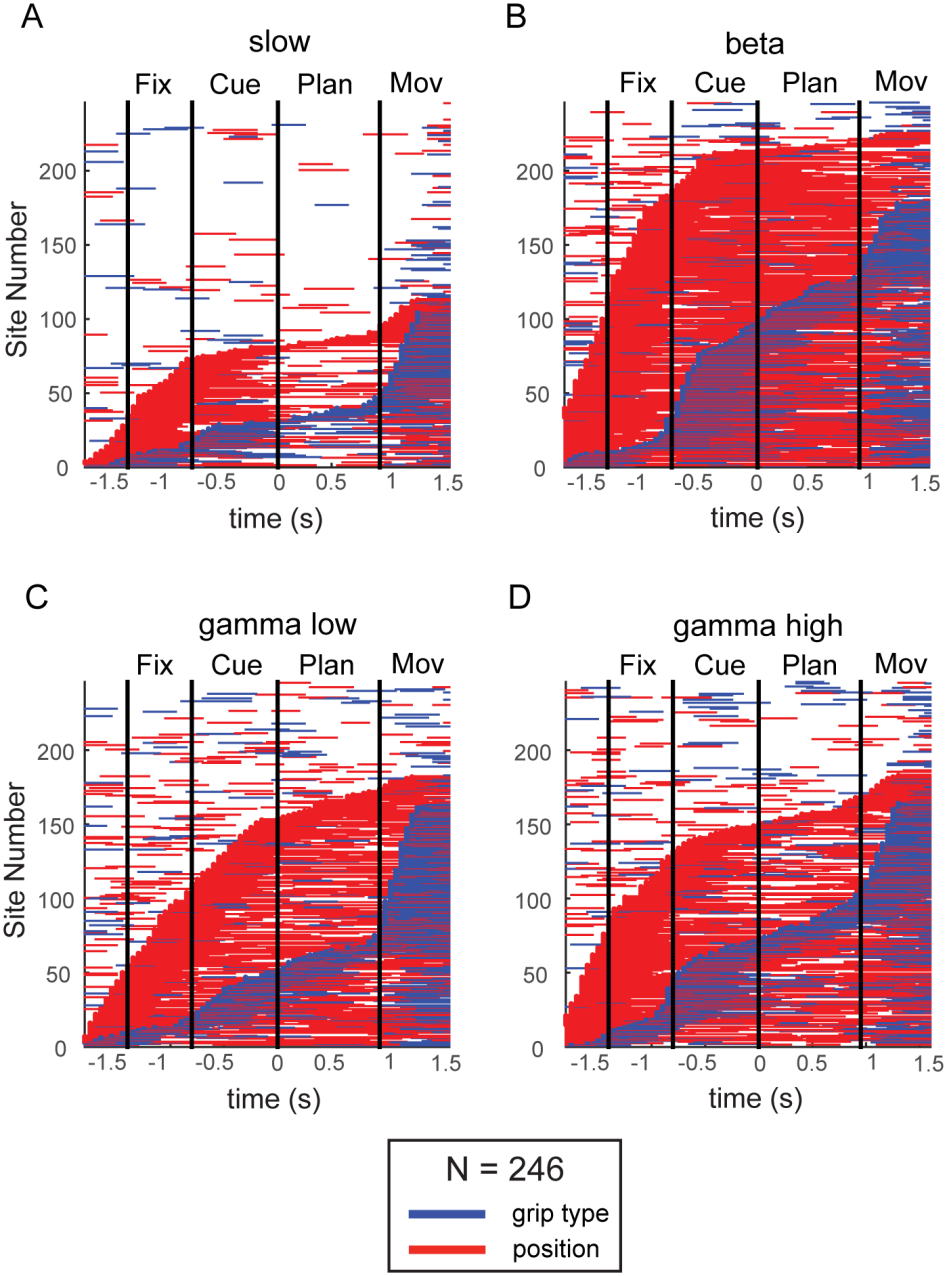
Tuning Onset. Sliding window analysis (window size 300ms, step size 50ms, two-way ANOVA, p < 0.05) in combined plots for both animals (N = 246 sites) revealed times with significant tuning in the slow frequency band (A), beta band (B), low gamma (C), and high gamma band (D). Horizontal lines indicate time periods with significant tuning for grip type (blue) and position (red), aligned to onset of the planning epoch. Sites are ordered by tuning onset (defined by the appearance of at least five consecutive significant steps). Vertical lines mark the border of the fixation, cue, planning, and movement epoch.

To gain a better understanding of the temporal progress of grip type and spatial tuning, we investigated the tuning onset of grip type and of spatial factors by performing a two-way ANOVA in a sliding window (length: 300 ms, shifted in steps of 50 ms), separately for each frequency band and recording site. Since results were similar across both animals, they are presented here together (Fig 4). Horizontal bars indicate time windows with a significant tuning for grip type (blue) or position (red), aligned to the start of the planning epoch. Each site is represented by a single row, and all sites are ordered (along the vertical axis) according to tuning onset, which was defined as the first occurrence of significant tuning for at least five consecutive windows.

For all frequency bands, grip type information (blue lines) generally started to exceed chance level in the cue epoch (best visible in the beta band), and rose continually and with a second peak in the movement epoch; before cue onset, the number of modulated sites were at chance level (p<0.05). In contrast, tuning onset of spatial position started generally earlier, during or even before the fixation epoch. This early tuning onset was likely due to motor noise while repositioning the target in the intertrial epoch in the dark, which has likely provided hints to the monkey about the upcoming target position before it was visually revealed in the cue epoch. In line with the previous tuning analysis for the different epochs, the strongest representation of both grip type and position was found in the beta band (Fig 4B). Furthermore, position tuning stayed persistent in the planning epoch in the beta and low gamma band (Fig 4B and 4C), whereas it was less persistent in the slow and the high gamma band (Fig 4A and 4D).

These findings are in line with a corresponding analysis of spiking activity in AIP [10], where position tuning also emerged earlier, remained more persistent, and in general was more prominent in the fixation, cue, and planning epoch than grip type tuning.

### Linear model

To further investigate the contributions of the different spatial factors to the overall spatial representations, we modeled the spectral activity of each frequency band and recording site for each task epoch as a function of grip type and the spatial position factors (i.e., target and gaze position). For this, we employed a stepwise linear model with the factors grip type, target position, and gaze position, which included horizontal and vertical coordinates for both target and gaze position (see Eq 2 in the Materials & Methods section). A site was considered to be modulated by target position or by gaze position, if at least one of its components (horizontal or vertical) was significantly contributing to the fit (p<0.05). This allowed us to analyze not only how many sites were modulated by spatial factors, but also to assess the specific contributions of these factors (target or gaze position), and in particular the tuning direction of these spatial components.

#### Significant model coefficients

**Fig 5:**
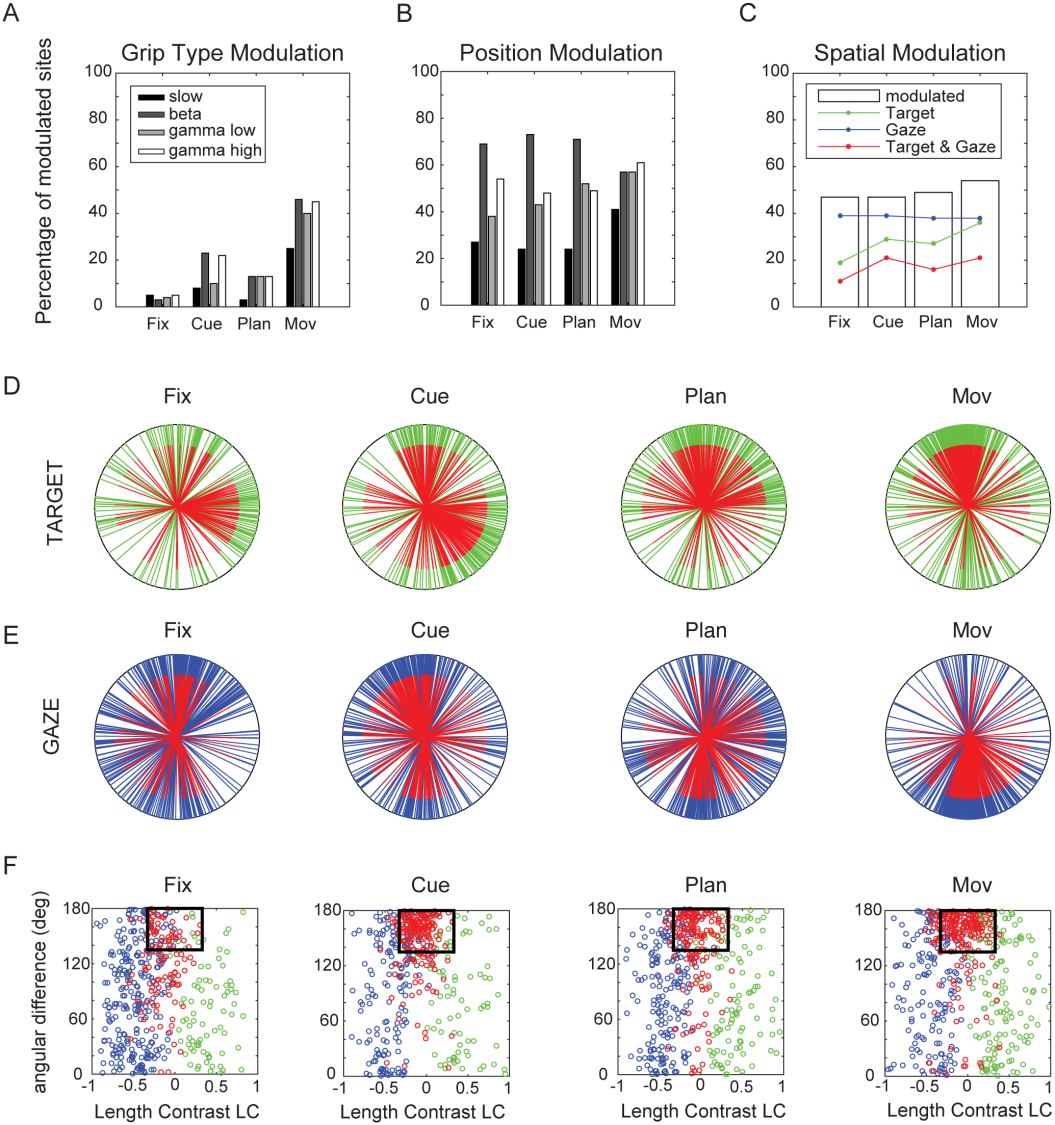
Linear model. A. Percentage of all LFP sites (both animals, N = 246) that have a significant coefficient for grip type in the various frequency bands (slow, beta, low gamma, and high gamma band) and task epochs B. Percentage of sites with a significant spatial position coefficient. C. White bars indicate percentage of sites with significant spatial coefficients averaged across bands and animals for the different epochs fixation, cue, plan, and movement. Colored lines indicate fraction of sites with spatial coefficients for target (green), gaze (blue), and for both (red). D. Directional tuning of target modulated sites (all bands) for the task epochs fixation, cue, plan, and movement, as revealed by the linear fit. Tuning directions are derived from the target coefficient vectors (green: target modulated; red: target and gaze modulated). E. Directional tuning of gaze modulated sites (blue: gaze modulated; red: target and gaze modulated). F. Scatter plots of spatially tuned sites illustrating angular orientation difference (y-axis) between target (green) and gaze position vectors (blue) against the length contrast (LC) of these vectors (x-axis). Sites with significant target and gaze modulation (red) were considered retinotopic if the coefficient vectors were of comparable length (| LC | < 0.33) and oriented in nearly opposite direction (angular difference < 135deg), as indicated by the black rectangles.

The percentage of significant coefficients of the linear model in each task epoch and frequency band is shown in Fig 5A and 5B across all recording sites. Results of both animals were similar (see: Table 1) and were therefore presented together. In agreement with the epoch-specific tuning analysis (2-way-ANOVA; Fig 3), sites with grip type representations in the linear fit increased for all frequency bands during the course of the task, i.e., towards movement execution (Fig 5A). In contrast, the fraction of sites with significant spatial coefficients was rather constant across the task epochs, and was largest in the beta band (Fig 5B), also in line with our previous analysis (Fig 3).

**Table 1:** Linear Fit—grip type and position modulation for different frequency bands.

	Grip type				Position			
BOTH ANIMALS (N = 246)	*fix*	*cue*	*plan*	*mov*	*fix*	*cue*	*plan*	*mov*
*slow*	5	8	3	25	27	24	24	41
*beta*	3	23	13	46	69	73	71	57
*gamma low*	4	10	13	40	38	43	52	57
*gamma high*	5	22	13	45	54	48	49	61
**animal P (N = 147)**								
*slow*	3	6	1	20	29	21	27	39
*beta*	3	34	9	48	59	63	61	45
*gamma low*	3	10	10	45	34	38	48	44
*gamma high*	5	21	12	45	59	54	47	54
**animal S (N = 99)**								
*slow*	8	10	5	31	24	27	20	43
*beta*	2	7	19	42	84	88	85	74
*gamma low*	4	11	18	32	44	51	58	77
*gamma high*	4	23	15	45	46	38	53	71

Fraction of recording sites (in %) with significantly coefficients for grip type or position, listed for different epochs (fixation, cue, planning, movement) and frequency bands (slow, beta, low gamma, high gamma). Percentages are listed for animal P (N = 147), animal S (N = 99), and both animals combined (N = 246).

#### Spatial factors contributing to specific activity modulations

Since the fraction of significant spatial coefficients of the linear fit was similar across frequency bands and animals (see Table 1), we present the further results in a combined fashion. For this, we pooled the data of both animals and all four frequency bands, which resulted in a combined dataset of 984 “sites” (4 frequency bands from 246 recording sites in two animals).

Fig 5C shows the percentage of all spatially modulated sites (i.e., averaged across animals and frequency bands) separately for the four task epochs. Open bars illustrate the fraction of sites that are modulated by at least one spatial factor, whereas colored lines depict the fraction of sites modulated by target (green), gaze (blue), and target and gaze combined (red). The contribution of gaze position remained relatively constant across all epochs (~40%) while the number of sites modulated by target position generally increased from fixation towards movement execution (from 19% to 36%, with a minimal drop during the planning epoch), suggesting a more prominent role of target position toward motor execution. Similarly, the fraction of sites modulated by both target and gaze position also generally increased during the task (from 11 to 21%). These trends were found in both animals, but spatial representations were generally larger in animal S (see Table 2).

**Table 2:** Linear Fit—grip type and spatial modulation (various factors).

**both animals (N = 984)**	*fix*	*cue*	*plan*	*mov*
*grip type*	4	16	10	39
*spatial*	47	47	49	54
*target (T)*	19	29	27	36
*gaze (G)*	39	39	38	38
*target and gaze (T&G)*	*11*	*21*	*16*	*21*
*T&G in Window (retinotopic)*	40	75	64	71
**animal P (N = 588)**	*fix*	*cue*	*plan*	*mov*
*grip type*	4	18	8	40
*spatial*	45	44	46	45
*target (T)*	17	24	20	29
*gaze (G)*	36	36	37	28
*target and gaze (T&G)*	*8*	*16*	*12*	*12*
*T&G in Window (retinotopic)*	45	68	65	68
**animal S (N = 396)**	*fix*	*cue*	*plan*	*mov*
*grip type*	5	13	14	38
*spatial*	50	51	54	66
*target (T)*	21	36	36	47
*gaze (G)*	43	43	39	53
*target and gaze (T&G)*	*14*	*28*	*21*	*34*
*T&G in Window (retinotopic)*	37	80	63	73

Fraction of recording sites (in %) with significant coefficient for grip type, any spatial position, and specifically for target, gaze, and both target and gaze, averaged across all frequency bands. Results are listed for different task epochs (fixation, cue, planning, movement) and for both animals separately and combined. Finally the percentage of all retinotopic sites (“in window”) is listed as a fraction of all target and gaze modulated sites.

#### Tuning direction

We also evaluated the coefficients of the linear model to determine the preferred direction of each spatially modulated site. Tuning directions, i.e. the normalized coefficient vectors obtained from the linear fit, were depicted in a radial plot for all sites modulated by target position (Fig 5D, green vectors) and gaze position (Fig 5E, blue vectors) for all bands together. Preferred directions of sites that were significantly modulated by target and gaze are also marked in red (Fig 5D and 5E).

Apparently, tuning directions were not homogenously distributed. Sites significantly modulated by target position (green) tended to have preferred directions towards the top in all epochs and towards the right or bottom-right position during fixation, cue, and movement planning. In contrast, sites significantly modulated by gaze position (blue) tended to be tuned upwards during fixation and cue instruction, then more to the top, right and bottom direction in the planning epoch, and finally strongly biased downward during movement execution. These diametrical changes of preferred direction suggest potentially different roles of these signals in visual (fixation & cue epoch) vs. motor-dominant (planning & execution) task epochs.

Furthermore, we directly compared the tuning direction of sites that were modulated by both target and gaze position (red vectors in Fig 5D–5E). Previously, it was reported that spiking activity in AIP contains strong retinotopic representations of target position, i.e., target position is encoded relative to gaze position (Lehmann & Scherberger, 2013). In order to see if that was also the case for LFP activity, we compared the coefficient vectors for target and gaze modulation with respect to their *tuning strength* and *tuning direction*. Tuning strength of both factors was compared by calculating a *length contrast index (LC)*, representing the relative length of the two coefficient vectors, whereas *the angular orientation difference* (Δ*φ*) measured the angle between the two vectors (see Materials and Methods).

Fig 5F shows the scatter plots of these two measures separately for each epoch. Sites that code only target (green) or gaze position (blue) show a LC close to +1 or -1, respectively. In contrast, sites that are modulated by target and gaze combined (and with similar vector lengths) have a LC close to 0 (red dots). Finally, LFP power of a given frequency band was considered to be retinotopic (i.e., coding target position in retinal coordinates), if their coefficient vectors for target and gaze had roughly similar length (LC close to zero: |LC|<0.33) and pointed approximately in opposite directions (absolute angular difference |Δ*φ*|>135°). Sites that fulfill these criteria fall within the black rectangles in Fig 5F.

The angular differences for both the target- and gaze-modulated sites were broadly distributed between 0 and 180 degrees during the cue, plan, and movement epoch (green and blue circles, respectively). However, as soon as the full spatial information was provided (cue epoch), more than two thirds of all sites that were modulated by target and gaze position (red dots) were located in the window indicating retinotopy (Fig 5F, Table 2). This fraction of retinotopically tuned sites was largest in the cue epoch (75%), but remained high also in the planning (64%) and the movement epoch (71%).

Interestingly, even though the fraction of retinotopically tuned sites remained constantly high throughout the task, the average tuning direction for target and gaze position dramatically changed: gaze representation, being predominantly tuned upwards in the cue epoch, switched over to the bottom position during the movement epoch (Fig 5E, red-blue). Similarly, target representation switched from bottom-right and top tuning to being dominantly tuned to the top position during the movement epoch (Fig 5D, red-green). The explanation for this effect is unclear, but might lie in the different character of the two epochs: dominant visual input in the cue epoch and motor preparation and execution in the movement epoch.

### Decoding Simulation

To assess the quality of LFP signals with respect to representing grip type and positional information, we performed an offline decoding analysis, in which we pooled the data from all recording sessions for each animal. On this dataset, we employed a Bayesian classifier to decode grip type and target and gaze position from the spectral LFP power. This decoding analysis was performed separately for each LFP frequency band as well as for all bands combined (for details see Materials and Methods section).

#### Grip Type Decoding

**Fig 6:**
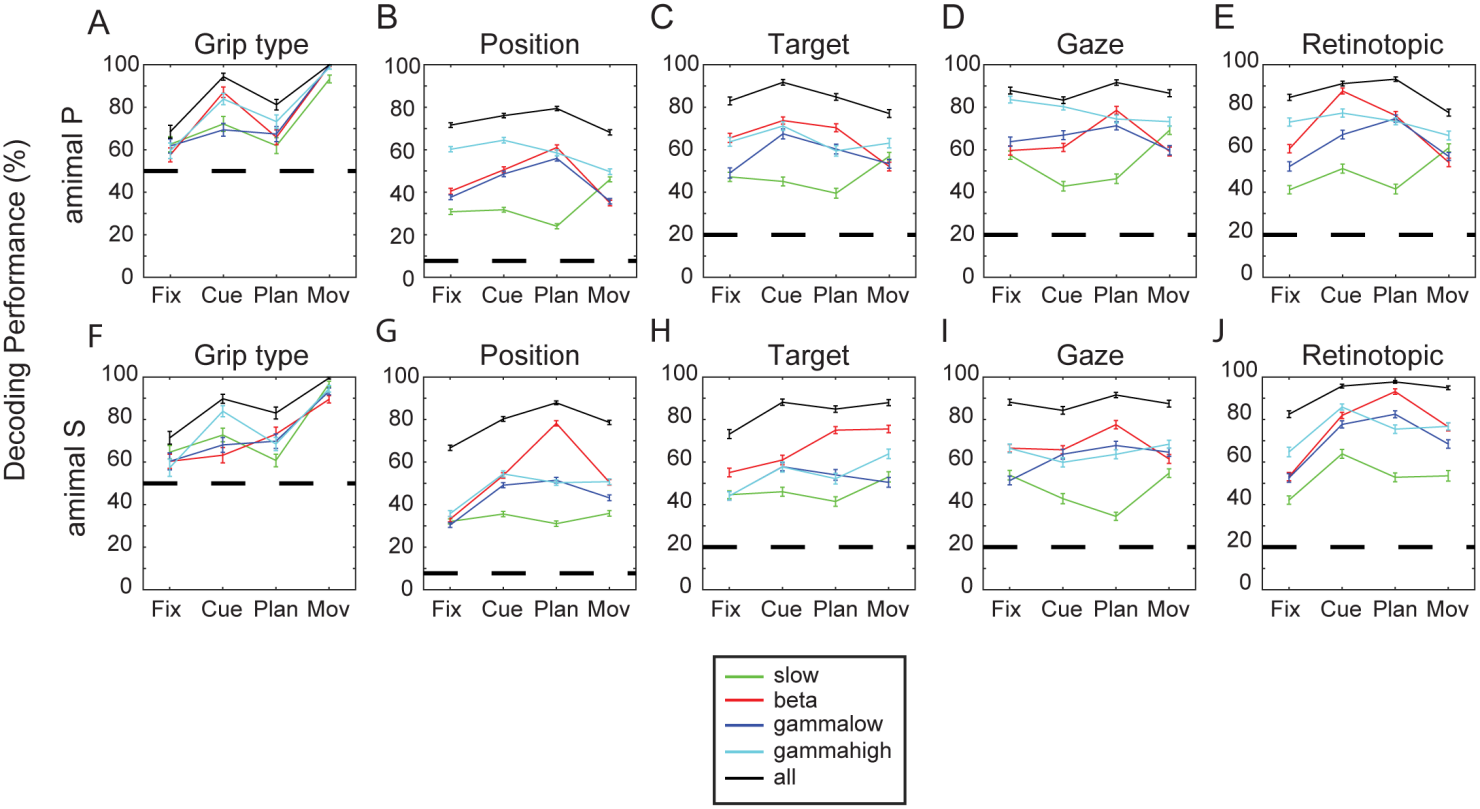
Decoding simulation. Simulated decoding performance of grip type and spatial factors for different frequency bands (slow: green, beta: red, low gamma: blue, high gamma: cyan, and all bands combined: black curves) and both animals (animal P: A-E, animal S: F-J). Individual panels show the decoding performance for grip type (A, F), the13 different spatial conditions (B,G), as well as for target (C,H), gaze (D,I), and retinotopic target position (E,J) separately for all task epochs. Dashed horizontal lines indicate chance level, and error bars the standard deviation after 100 simulated decoding repetitions.

In the single-site analysis presented above, grip type representation was strongest during the movement epoch (Figs 3A, 3C and 5A). It was therefore not surprising that decoding of grip type was best in the movement epoch. In both animals, decoding performance was generally above 90% when decoding from individual frequency bands and close to 100% when all bands were combined (chance level 50%; see Fig 6A and 6F). In the earlier task epochs (cue and planning), decoding performance was somewhat lower, ranging between 60–80% for individual frequency bands and above 80% for all bands combined (Fig 6A and 6F). These decoding results confirmed our findings on LFP grip type selectivity (Figs 3–5) and are in agreement with corresponding decoding results based on spiking activity [6, 27].

#### Position Decoding

For the decoding of position information, performance was best when considering all frequency bands combined. Performance reached values between 76 and 88% in the cue and planning epoch for both animals (black curve; chance level for 13 spatial conditions: 7.7%; Fig 6B and 6G). For decoding with individual frequency bands, performance was lowest for the slow band (~30%), whereas the other bands (beta, gamma low, gamma high) showed their maximal performance in the cue or planning epoch (Fig 6B and 6G).

When decoding individual spatial components (target position, gaze position, or retinotopic target position; Fig 6C–6E and 6H–6J), performance levels were above 80% when decoding from all bands together (chance level 20%), and even reached values above 90% for retinotopic decoding in the cue or planning epoch (Fig 6E and 6J, black line). Decoding with individual frequency bands was again lower, with the beta frequency band tending to perform best and the low frequency band worst (Fig 6C–6E and 6H–6J).

In summary, our analyses revealed a substantial representation of both grip type and spatial information in the LFP of AIP, with an increase of grip type representation toward movement execution and a rather constant encoding of spatial information during the task. Several independent analyses demonstrated that each of these spatial factors (target, gaze, and retinotopic target position) was represented in the observed LFP frequency bands and could be reliably decoded.

## Discussion

We analyzed local field potentials in AIP during the planning and execution of a reach-to-grasp task, in which target and gaze position were systematically varied. In all of the analyzed frequency bands (slow [1–13 Hz], beta [13–30 Hz], low gamma [30–60 Hz], and high gamma band [60–100 Hz]), we found representations of grasp type, not only restricted to the movement epoch, but also during movement instruction and preparation. Grasp preferences were strongly biased to either precision or power grip, which, however, strongly varied between animals and frequency bands (Fig 3A and 3C). Besides the encoding of grasp type, we found representations of spatial factors throughout the task, consistent with previous results from spiking activity [10]. Despite the different number of conditions for grasping and for target and gaze position, we argue that the neural selectivity for these factors remains comparable, since power and precision grips range on opposite sides of a spectrum of possible grip types, both in terms of kinematics and neural representation [6], whereas the variability of the spatial conditions is limited by its directional tuning (Fig 5).

Both tuning and linear regression analysis ascribed the strongest representation of spatial information in the beta frequency band (Figs 3B, 3D & 5B). The linear fit further revealed significant contributions of both target position and gaze position to these spatial representations, with a solid fraction of sites modulated by both factors (Fig 5C). Of these, a considerable fraction of sites (cue: 75%, plan: 64%, mov: 71%) encoded information in a retinotopic fashion, defined by target and gaze vectors being tuned in approximately opposite directions (Fig 5F). Furthermore, decoding simulations of grip type confirmed these findings, with best performances for the movement epoch (Fig 6A and 6F), whereas decoding of spatial factors from single frequency bands revealed similar performances for the beta, low, and high gamma bands, which were clearly higher than for the slow band. This was the case for decoding all 13 different spatial conditions combined (Fig 6B and 6G) as well as for predicting target position (Fig 6C and 6H), gaze position (Fig 6D and 6I), or retinotopic target position separately (Fig 6E and 6J). As expected, decoding performance was always maximal when all frequency bands were combined.

These results demonstrate that neural representations of grasp type and spatial information is not restricted to single units in AIP [10], but is also found in the LFP activity both during grasp planning and execution. Results are consistent with previous LFP studies of parietal, premotor, and motor cortex, that have reported the encoding of sensory and motor related signals during the planning and execution of visually guided actions [18–21].

### Grasp representation

AIP is known to be involved in the sensorimotor transformation related to grasp movements [1–5]. It is therefore not surprising that we found a strong modulation of LFP activity during movement execution. In other motor areas, strong increases in spectral power have been reported after changes in behavioral state [18, 28] or during object hold [25, 29]. Interestingly, we found in all frequency bands a strong predominant selectivity for one of the two grip types during movement execution, which was consistent across frequency bands in one animal (Fig 3A), but not the other (Fig 3C).

Similar to our findings, previous studies have also reported strongly biased LFP signals with respect to grip type preferences. For example, Asher et al. reported strong modulations of LFP activity in the intra-parietal lobule (IPL; combined AIP and area 7b) during the movement phase of a reach-to-grasp task and found a general preference for power grips in two tested animals [24]. Similarly, a study of LFP activity in the premotor hand grasping area F5 and in motor cortex (M1) reported strong selectivity for grip type in the beta frequency band, in particular towards the end of grasp movements during the so-called “hold” epoch [25]; this selectivity between six different grasp types was strongly biased towards encoding of a hook grip. Furthermore, the same study showed a strongly biased frequency-dependent selectivity, which was only partially consistent between animals [25]. These findings were similar to our observations in animal S and might hint on a general bias for certain grip types in motor areas, potentially specific for individual animals. Our task design involved only two grip types, with differently biased preferences for different animals and frequency bands. It therefore remains to be determined how well LFP signals could contribute to a robust decoder of many grip types, e.g., as needed for an LFP-based brain machine interface [23, 30, 31].

### Spatial representations

In the LFP, we also found representations of target position, gaze position, and retinotopic target position, in all task epochs and across all tested frequency bands, being strongest in the beta band (Figs 3B, 3D and 5B), and we could reliably decode these spatial positions from the LFP throughout the task (Fig 6B–6E and 6G–6J). Representation of reach direction in the LPF of AIP has been reported before [24]. However, in contrast to our results, they found the highest representation in the slow band and during the cue and movement epoch, but not in between, i.e., during movement planning. These differences could originate from different task designs: our animals reached for target positions on a vertical board, while their monkeys performed center-out reaches on a horizontal plane, resulting in movements towards or away from the body.

Furthermore, our task setup allowed us to vary gaze position, which facilitated a closer and more specific look at both factors contributing to coordinated eye-hand-movements: target and gaze position. The higher representation of gaze position compared to target position throughout the task (Fig 5C, blue & green curves) was in line with our findings from the single neuron population in AIP [10]. The increase of target representation from fixation to cue epoch (after target position was illuminated) as well as from plan to movement epoch suggests a task-relevant representation of target position.

The non-uniform distribution of directional preferences for both target and gaze representations (Fig 5D and 5E) seems to be a general feature of LFP signals, which has been repeatedly reported for several visuo-motor and motor cortical areas [18, 22, 24, 32]. Interestingly, this tuning bias strongly varied over the course of the task, both for target and gaze position. Gaze position preference switched from the top position during the fixation and cue epoch to the bottom position during movement, whereas target preference shifted to the top position during movement. This might be due to the different nature of the two task epochs, changing from a strongly visual representation during the cue epoch to a motor representation during the movement epoch. Interestingly, we also found such a tuning bias for specific reach directions when exploring single unit spiking activity [10]. However, their tuning preferences remained rather constant over time, suggesting that different fractions of single units contributed to the LFP across different task epochs.

Also similar to spiking activity [10], a considerable fraction of LFP sites in AIP was modulated by both target and gaze position. For the majority of these sites, we demonstrated retinotopic tuning characteristics after cue onset (Fig 5F, Table 2). Similar retinotopic representations of reach direction have been reported in spiking activity of other parietal areas like V6A [33], the parietal reach region [19, 34–37], and the parietal area 5 [35, 38], however, to our knowledge, not for LFP activity.

In summary, this study found a strong representation of spatial information in the LFP of the grasping area AIP, including target position, gaze position, and target position in retinotopic coordinates. Unlike the representation of grip type, which was predominantly found during movement execution, spatial representations were present in the LFP throughout the task. A decoding simulation demonstrated that both grip type (in particular during the movement epoch) and spatial positions can be decoded highly accurately. These findings might be relevant for the development of LFP-based neural interfaces for motor control.

## Supporting Information

S1 Checklist Arrive Checklist. (PDF)
(PDF)
